# Adolescents with orofacial clefts: understanding their experiences

**DOI:** 10.1590/1984-0462/2024/42/2023131

**Published:** 2024-05-06

**Authors:** Marina Gifalli, Camila Trettene Antonio, Verônica Aparecida Pezzato da Silva, Francine Aroteia Capone, Priscila Capelato Prado, Armando dos Santos Trettene

**Affiliations:** a Universidade de São Paulo, Bauru, SP, Brazil.

**Keywords:** Cleft lip, Cleft palate, Adolescent, Qualitative research, Nursing, Fenda labial, Fissura palatina, Adolescentes, Pesquisa qualitativa, Enfermagem

## Abstract

**Objective:**

To understand the experience of young people with orofacial clefts regarding life as an adolescent.

**Methods:**

Descriptive, qualitative study, developed in a Brazilian public and tertiary hospital, a reference center in the care of patients with craniofacial anomalies and related syndromes, between February and April 2019. The sample was defined by theoretical saturation. The following inclusion criteria were established: age between ten and 19 years old and having previously operated on orofacial cleft (lip and/or palate). Individuals with fissure associated with syndromes or other malformations were excluded. Data collection was performed through semi-structured interviews, which were audio recorded and transcribed in full. The trigger element was: how has it been for you to experience your adolescence? For the construction of the results, content analysis was used in the thematic modality.

**Results:**

Seventeen adolescents participated. From the speeches, three categories were revealed: interacting socially, feeling supported, and experiencing and facing prejudice.

**Conclusions:**

The biopsychosocial and conflicting complexity that adolescents with orofacial clefts experience was noticed, as well as the importance of receiving support and establishing modalities of situational coping.

## RESUMO

**Objetivo:**

Compreender a experiência de jovens com fissura orofacial quanto à vivência da adolescência.

**Métodos:**

Estudo descritivo, qualitativo, desenvolvido em um hospital público e terciário brasileiro, referência no atendimento de pacientes com anomalias craniofaciais e síndromes relacionadas, entre fevereiro e abril de 2019. A amostra foi definida por saturação teórica. Estabeleceram-se como critérios de inclusão: idade compreendida entre dez e 19 anos completos e apresentação de fissura orofacial (lábio e/ou palato) previamente operada. Excluíram-se aqueles que apresentavam a fissura associada a síndromes ou outras malformações. A coleta de dados foi realizada por meio de entrevista semiestruturada, que foi gravada em áudio e transcrita na íntegra. O elemento disparador foi: como tem sido para você vivenciar sua adolescência? Para a construção dos resultados, utilizou-se a análise de conteúdo na modalidade temática.

**Resultados:**

Participaram 17 adolescentes. Com base nos discursos, desvelaram-se três categorias: interagindo socialmente; sentindo-se apoiado; e vivenciando e enfrentando o preconceito.

**Conclusões:**

Percebeu-se a complexidade biopsicossocial e conflituosa que adolescentes com fissura de orofacial vivenciam, assim como a importância de eles receberem apoio e de se estabelecerem modalidades de enfrentamento situacional.

## INTRODUCTION

Orofacial clefts stand out among the malformations that affect the face, with a prevalence of 1:650 live births in Brazil. The multifactorial etiology includes both genetic and environmental factors. They occur in isolation or in association with syndromes and other malformations, affecting the lip, alveolar ridge and/or palate.^1,2^ They can cause functional, aesthetic and psychosocial implications. When babies, the functional implications are in evidence, however, in childhood and especially in adolescence, the psychosocial and aesthetic aspects are experienced in greater proportion.^3,4^ In fact, in adolescence, in addition to physical changes, there is a need for social and affective interaction and enhancement of aesthetic standards. Thus, adolescents with clefts may experience feelings or situations of rejection and discrimination, impaired self-esteem, depressive symptoms, anxious behavior, low acceptance, changes in self-concept and self-image, difficulty in social interaction, among others, culminating in a lower quality of life in addition to more prone to mental and/or psychological disorders.^5,6^

Different studies carried out with children and adolescents with orofacial clefts are available. Among them, a quantitative investigation found depressive symptoms in children and adolescents with orofacial clefts, aged between seven and 17 years, in a geographically localized Brazilian population, with no statistically significant difference compared to those without clefts.^7^ Among 52 children and adolescents aged seven to 15 years old, of both sexes, with cleft lip and palate or isolated palate, with and without associated hearing loss, all experience the hardship of living with the aesthetic and functional impairment caused by anomaly and living in a society that values image and judges differences.^8^ In another research with a quantitative approach, it was shown that body image and quality of life are influenced by perceptions of appearance, although they are similar to those of young people without the malformation.^6^ Another study, which included 78 adolescents with cleft lip and palate aged 15 and 18, showed that among those with less intelligible speech, their peers showed more negative attitudes, including cognitive, affective and behavioral.^4^ Furthermore, early surgical intervention significantly influenced the speech, physical and mental health and social inclusion of Indian children and adolescents.^3^ Another investigation, carried out with 22 pre-adolescents aged between nine and 13 years old, aimed to identify the main stressors related to orofacial clefts, as well as how to cope.^9^ Other researchers had the specific objective of verifying the criteria that cleft teenagers establish to select romantic partners and whether these differ from those used by those without the malformation. Adolescents aged between 14 and 21 years old, of both sexes, participated. There was no significant difference in the choice of romantic partner, although there was an influence on the view of oneself and others in this process.^10^

The correlation between religiosity, spirituality and quality of life was evaluated in a case-control study, whose case group consisted of 40 adolescents with unilateral cleft lip and palate, aged 15-18 years old, which showed the non-existence of a relationship between variables for most aspects evaluated.^11^ A similar result was observed in another study, in which a hundred adolescents participated (50 with unilateral cleft lip and palate and 50 bilateral). Although the patients had high levels of religiosity, spirituality and self-esteem, no correlation was observed between the variables.^12^ The prevalence of smoking was 20.6% among adolescents with orofacial clefts, aged between 12 and 19 years, being influenced, among others, by the presence of cleft lip and palate.^13^ The qualitative approach was also used in research that aimed to understand the experience of children and adolescents with clefts, understand their needs and how to act to provide them with clarification and guidance. Nine individuals aged between seven and 18 participated. It was noticed that the implications of the malformation influence the health of the child/adolescent, the family and society, and may compromise psychosocial adjustment. It was also revealed that rehabilitation and psychological maturity help with resilience.^14^

In summary, the available investigations present great heterogeneity regarding the age of the participants, in addition to the specificity of the topics covered, the vast majority of which have a quantitative approach. Thus, gaps are evident in the understanding of how adolescents with orofacial clefts experience adolescence, mainly with the use of qualitative methodologies, in which the main objective is to develop a deep understanding of a subject, from the individual’s perspective.

It seems that the coping modalities used by these adolescents significantly influence the way they perceive and face difficulties, as well as how they establish family, social and cultural relationships.^11-13^ In this context, we sought to answer the following question: how do young people with orofacial clefts experience adolescence? Considering the vulnerability of these adolescents in relation to aesthetic, psychological, social and communication problems, it becomes essential to reveal these experiences, in the hope of promoting interventions and public policies. In this sense, the objective was to understand the experience of young people with orofacial clefts regarding adolescence.

## METHOD

This is a descriptive study, with a qualitative approach, built from the Consolidated Criteria for Reporting Qualitative Research (COREQ),^15^ developed between February and April 2019, in a Brazilian public hospital of tertiary level, specialized in the care of people with craniofacial anomalies and related syndromes.

Adolescents aged between ten and 19 years old and with orofacial clefts (lip and/or palate), uni or bilateral, of both sexes, previously operated on, were invited to participate. Those who had the fissure associated with syndromes or other malformations were excluded, in addition to those who were using psychotropic drugs, undergoing psychiatric and/or psychological treatment.

At the institution, the setting for this research, an average of 40 adolescents are served monthly. Of these, approximately 27 met the inclusion criteria established for this study. However, the intentional and convenience sample was defined by theoretical saturation, that is, when the statements became recurrent, and no new elements were highlighted.^16^ During data collection, which was consecutive, four adolescents refused to participate and theoretical saturation occurred in the 17^th^ interview, that is, 17 adolescents participated.

Previously, the adolescents were invited to participate in the research and explained about the objectives and implications of the study in clinical practice. Data collection was carried out exclusively by the main researcher, who was previously trained, and by her advisor, who has experience in qualitative studies. Both are nurses and had no direct contact with the participants.

For this, a semi-structured interview was used based on the trigger element: how has it been for you to experience your adolescence? The approach was individualized and carried out in a private environment, which lasted 20 minutes on average, being audio recorded and later transcribed in full. At the end of each one, the recording was shown to the adolescent, and he was asked about his desire to remove or add something. No new approaches were needed and no modifications were made. In addition, the participants were characterized in terms of age, sex, marital status, having children, socioeconomic status^17^ and education.

After the transcriptions, the content analysis methodology was used in the thematic modality, in which the stages of pre-analysis, exploration of the material and interpretation were covered. Initially, a fluctuating reading of the contents of the interviews was carried out, observing the principles of pertinence, exhaustiveness, homogeneity and representativeness. Sequentially, the coding operations were constructed in thematic categories, through the identification of keywords and related themes. Finally, the treatment of the results was obtained by inference and interpretation of the contents, supported by the foundation and justification of the study.^16^ This process occurred at the end of each interview, aiming to identify theoretical saturation. At the end of the study, the results were presented to the adolescents during their return for care at the institution that was the research setting.

The study was approved by the Research Ethics Committee of the Hospital through Certificate of Presentation for Ethical Appreciation (CAAE): 04295718.6.0000.5441, and all current ethical precepts were observed. To ensure the anonymity of the participants, the letter "A" for teenager was used to identify the speeches of each individual, plus sequential Arabic numerals.

## RESULTS

The participants were 17 adolescents, with an average age of 15 years (±2.3), eight of whom were male (n=9; 53%). Regarding the type of orofacial cleft, ten had unilateral cleft lip and palate, three had unilateral cleft lip, two had bilateral lip and palate cleft and two had cleft palate. All underwent lip and/or palate reconstruction surgeries until the age of two, in addition to secondary surgeries, including nasal and orthodontic rehabilitation, according to the indication. All were single and had no children. As for social class, the low class prevailed (n=11; 65%), with incomplete secondary education (n=9; 47%).

From the speeches, three categories were listed:

1. Interacting socially;
2. Feeling supported;
3. Experiencing and facing prejudice.

## Interacting socially

In this category, it was possible to understand about social interaction, including behaviors, actions and experiences. One of the most important aspects for social interaction is communication, which, due to the fact that the cleft affects the oral cavity, will be impaired. These difficulties were noticed and included the fear of public speaking, in addition to negative feelings, such as embarrassment or shame.

*"Some people ask why I speak that way [...] I don’t know how to answer [...] I don’t like to read and present work at my school, because I don’t like to expose myself [...] Everyone respects me, but I do not feel comfortable."* (A15)

*"Some people sometimes don’t understand what I’m saying because of my nasal voice [...] It bothers me a lot! I prefer to remain silent."* (A9)

However, it was noticed that the difficulties experienced were resolved or minimized during the rehabilitation process.

*"When I was younger, I had difficulty communicating [...] people didn’t understand [...] but now it’s easier for them to understand me, because my voice got better after the surgeries and speech therapy."* (A6)

*"Communicating has never been a big problem for me, even if at times people don’t understand me."* (A8)

Another means of social interaction was related to the use of social media, utilized for relationships or to discuss subjects they have an affinity or identify with. However, although the adolescents with orofacial clefts in this study mentioned using them, it was noticed that they did not like to publish their photos, which may be related to dissatisfaction with their appearance or aesthetic standards.

*"I have WhatsApp and Facebook [...] I like to post about video games and share topics about it... but it’s rare for me to post pictures of myself [...] I don’t like to be seen!"* (A16)

The use of social media for love relationships was also noticed. Although this modality of dating is currently in evidence, it can reveal the difficulty of adolescents with a fissure in interacting face to face. On the other hand, one participant pointed out the absence of obstacles related to the love relationship.

*"I date long distance [...] We’ve never met in person [...] we’re going to complete five months together [...] It took a while, but I found a nice girl who understands my difficulties [...] I always think about what it will be like when we meet."* (A9)

*"I’ve been dating for a month today. I’ve known him for three years, he’s my best friend at school. Things happened naturally. We met, interacted and our friendship turned into love."* (A10)

### Feeling supported

It was noticed in this category that, with the rehabilitation process, the participants showed feelings of gratitude, protection, and support, based on family, church, friends and the hospital institution where they are treated. It was learned that the family is synonymous with support, especially in the most difficult moments, such as, for example, during surgeries and in the postoperative period.

*"My family is always by my side supporting me, especially when I am about to have surgery or I am recovering from one."* (A17)

*"I am very grateful to my family for always being present in my life, giving me support, especially in relation to my treatment."* (A4)

The church was also revealed as an important source of spiritual, emotional and even financial support. It was noticed, additionally, that faith is not limited to attending churches, as some participants preferred to say their prayers at home.

*"The church is like my second home. I’ve been going there for ten years [...] so I know most of the people and have many friends. They help me a lot, psychologically, spiritually and even financially."* (A8)

*"I have faith, but I don’t go to church much. I prefer to pray at home, but I cannot imagine myself without my God!"* (A13)

*"My family goes to church. I am not a practitioner, but I have my faith and I believe that it helps me a lot to overcome my difficulties." (A14)*

As for friendships, it was noticed that the adolescents did not have difficulties in building them.

*"When I was younger, it was difficult for me to make friends. I have a friend I’ve known for eight years [...] he was the one who welcomed me and included me in the group of friends [...] now I can make new friends myself."* (A6)

*"I have a lot of friends, mainly because of skateboarding, which I started training with my neighbors when I was little, and we always hang out together."* (A11)

Another source of support/disclosed support was the hospital institution where the adolescents had treatment done, and this is evidenced in their contact with other patients with clefts of different complexities.

*"When I come to the hospital that has treated me since I was a baby, I realize I’m not alone and I’m not the only one with limitations [...] I know other people with problems that are even more serious than mine and that helps me to face my difficulties."* (A2)

*"Here at the hospital, I feel welcomed [...] I know other children and adolescents who have bigger problems than mine [...] I feel that I am not alone."* (A5)

### Experiencing and facing prejudice

Adolescents with clefts experience situations and moments where it is difficult to cope, including those related to bullying by classmates in the school environment, aesthetic issues and speech disorders. Family and friends were cited as protective factors at these times.

*"My colleagues look at me differently and call me nicknames [...] This makes me very sad."* (TO 1)

*"At my old school I was bullied, but my friends always helped me to deal with it. Many people saw and did nothing. In this new school, I don’t suffer from this, but if it happens, it doesn’t matter either, I feel more prepared to deal with bullying, because I have support from friends and my family."* (A12)

*"I hear a lot of mean jokes from my colleagues [...] I never gave them much credit; I’ve always been happy [...] by myself."* (A10)

It was also apprehended that some participants, although experiencing situations characterized as bullying, did not perceive them as such, even though they included looks and indiscreet questions.

*"I realize that people look at me differently [...] I think it’s out of curiosity, maybe [...] Some ask indiscreet questions, but I’ve never been bullied, thank God."* (A3)

*"When I was younger, I heard jokes from colleagues, but I believe I wasn’t bullied, they were just jokes."* (A1)

## DISCUSSION

It was revealed, in the present study, that some adolescents felt uncomfortable with communication, since sometimes they spoke and were not understood by their peers. This fact is extremely important, since social interaction begins with communication. In other words, this aspect is very important for the development of early childhood and the strengthening of interpersonal relationships among adolescents, since, through speech, social and affective ties are established, personality is enhanced, new knowledge is constructed and experiences are shared.^8^

Although the first surgeries to repair orofacial clefts are performed in childhood, including palatoplasty, communication disorders may occur which are related to the failure of the surgery or its complications, including the presence of fistulas or velopharyngeal dysfunction that cause the passage of air from the oral cavity to the nose during speech. In addition, hearing and dental alterations may occur,^18^ which add to the problems related to communication.

However, it was possible to apprehend that the functional difficulties of the participants, including speech, significantly improved with the rehabilitation process. On the other hand, an investigation involving young people aged between seven and 18 years old with orofacial clefts pointed out they had anxiety, stress and frustration, associated with moments of embarrassment during their relationships.^14^

Another context revealed in this study showed that social media have become one of the main means of communication among young people in the contemporary world, facilitating social interaction. A qualitative study pointed out that they provide knowledge, becoming a source of quick, easily accessible information and facilitating the building of friendships, in addition to favoring an environment of free expression. In contrast, some adolescents pointed out social isolation, exposure to personal life and vulnerability to violence and abuse as a negative factor.^19^

In the present study, the adolescents stated that they use social media to post content that they like or are interested in. However, they reported not displaying their own photos or texts. Another unveiled issue referred to love relationships through social media, without meeting in person. These reports may be related to aesthetic and communication problems, which influence self-esteem or self-image.^4,20,21^

The development of love relationships in adolescence stands out, as it is at this stage of life that discoveries and affective interests begin. In this sense, a Brazilian study that compared a group of adolescents with clefts to a group without them, regarding the moment of choice of partners, identified that there were no significant differences between both groups.^9^

However, adolescents with orofacial clefts may have psychosocial problems, including depressive symptoms.^7^ Thus, the benefits of support networks as a method of coping and overcoming are evident. In this study, feelings such as gratitude, support, and acceptance by the family or friends were disclosed. In fact, having an active and healthy social relationship with family, friends and in the school environment favors the process of social inclusion, the construction of dreams and acceptance of one’s physical condition.^14,22^

Considering the long rehabilitation process of orofacial clefts, adolescents tend to become attached to people who help them solve problems associated with the treatment and offer of emotional support, especially family members. In this sense, the main mode of coping is acceptance.^10^

Another coping modality referred to religiosity and spirituality, which the participants pointed out as an important factors to overcome difficulties and negative experiences. In fact, adolescents with clefts that have higher levels of religiosity and spirituality have a better perception of quality of life and deal better with adversities, in addition to having healthier lifestyle habits.^11-13^

The benefits of religiosity and spirituality among adolescents include, among others, a reduction in anxiety and depression, an increase in satisfaction and quality of life and a decrease in stress as a protective factor against the risks of psychosocial vulnerability such as the use of licit and illicit drugs.^23-25^

In addition, living with other patients in the hospital environment was perceived as a way of coping. The rehabilitation process is long, lasting an average of 18 years, and several surgeries are performed.^26^ During hospitalizations, adolescents exchange experiences, in addition to seeing that they are not the only ones affected by the malformation.

The main difficulties experienced by the adolescents that were revealed in this study were related to social interaction, especially in the school environment, including bullying, an offensive practice that can cause serious harm to victims by affecting their psychological and physical integrity, predisposing them to low self-esteem, depressive symptoms, fear, anxiety, among others.^27^

In this direction, a survey that included 41 young people with unilateral cleft lips and palates, aged between eight and 16 years, showed that about 30% reported having been frequent victims of bullying at least two to three times a month, while another 32% were bullied one to two times in the last two to three months.^28^

Bullying is strongly related to risk behaviors, such as alcohol use, illicit drugs, involvement in fights, among others, where the emotional and psychological consequences experienced in adolescence tend to extend into adulthood. Thus, it is essential to guide adolescents with regard to bullying and its consequences, considering that many young people interpret such practices as jokes,^28,29^ as evidenced in this study.

Individuals with orofacial clefts have low levels of self-esteem compared to unaffected individuals, as evidenced in a survey involving 160 individuals aged 12 to 50 years old.^30^ Currently, body image represents personal identity, relating to feelings, thoughts and behaviors about one’s own body, which can be influenced by friends, the media, family, peers, social media, among others. In this context, the adaptive process is pointed out as a coping strategy that allows the individual to face their choices and is associated with the support offered.^10^

Furthermore, it must be considered that women express greater dissatisfaction with their appearance compared to men,^30^ a fact that may have influenced the findings of this investigation, whose participants were, for the most part, men. However, it is relevant that the self-image and/or self-esteem are related to objective and subjective factors, not being directly related to the presence or complexity of the orofacial cleft, but rather, the importance given to it, in addition to the establishment of situational coping modalities.^12,14,19^

Finally, approaching adolescents with cleft lips and/or palates in a single moment may not have been sufficient to understand all the nuances and meanings attributed to the experience of adolescence, and this represents a limitation. Furthermore, the participation of adolescents aged, on average, 15 years old limited, for example, their experiences regarding love relationships, including dating. Another fact to be considered refers to the fact that some of the participants have not yet entered adolescence. Therefore, further investigations are necessary to expand the findings.

However, the benefits of this investigation are evident, and its main contribution refers to the identification of coping methods, as well as the support network experienced by adolescents, based on the experience of the functional and psychosocial implications related to orofacial clefts.

It was revealed that adolescents with orofacial clefts had difficulties in social interaction related to communication, mainly due to hypernasality, culminating in negative feelings such as shame and embarrassment. Although they use social media, the fact that they do not post photos or have romantic relationships virtually could reveal difficulties in social interaction and body image.

Bullying stood out among the difficulties, even in situations where it was not interpreted as such, such as in the school environment. However, the support shown by family and friends and during treatment as well as contact with other young people with similar problems contributed significantly to overcoming the difficulties experienced. The following sources of support were revealed: the family, in particular, followed by religiosity and/or spirituality, friends and the hospital institution where the rehabilitation process was conducted. In short, the findings point to the biopsychosocial and conflicting complexity that young people with orofacial clefts experience in their adolescence, deserving special attention from all those involved in this process.

## Data Availability

The database that originated the article is available, with the corresponding author.
